# A Protest against Pasteurism

**Published:** 1889-07-27

**Authors:** John Roche


### A Protest Against Pasteurism.

By John Roche, M.D.

Your observations on hydrophobia are most opportune. Pasteur treatment and institution are quite in the abstract as ridiculous as a McGovan's. There is disinterested testimony that McGovan has cured hydrophobia. There is none that Pasteurs has, and there is quite as good evidence in favour of McGovan's prevention as there is in favour of Pasteur's.

From analogy, hydrophobia being like the tetanus induced by alcoholism, which has been cured by mustard baths and Jaborandi diaphoretics, I would be inclined to believe that the same might happen in hydrophobia.

If the McGovans cure hydrophobia by diaphoresis, I may hope the secret is worth studying, and superior to what is published.

